# The effective deficiency of biochemical networks

**DOI:** 10.1038/s41598-023-41767-1

**Published:** 2023-09-04

**Authors:** Damoun Langary, Anika Küken, Zoran Nikoloski

**Affiliations:** 1 Bioinformatics, Institute of Biochemistry and Biology, University of Potsdam, Potsdam, Germany; 2 Systems Biology and Mathematical Modeling, Max Planck Institute of Molecular Plant Physiology, Potsdam, Germany

**Keywords:** Computational biology and bioinformatics, Biochemical reaction networks, Computational models, Metabolic engineering, Computer modelling, Metabolomics

## Abstract

The deficiency of a (bio)chemical reaction network can be conceptually interpreted as a measure of its ability to support exotic dynamical behavior and/or multistationarity. The classical definition of deficiency relates to the capacity of a network to permit variations of the complex formation rate vector at steady state, irrespective of the network kinetics. However, the deficiency is by definition completely insensitive to the fine details of the directionality of reactions as well as bounds on reaction fluxes. While the classical definition of deficiency can be readily applied in the analysis of unconstrained, weakly reversible networks, it only provides an upper bound in the cases where relevant constraints on reaction fluxes are imposed. Here we propose the concept of effective deficiency, which provides a more accurate assessment of the network’s capacity to permit steady state variations at the complex level for constrained networks of any reversibility patterns. The effective deficiency relies on the concept of nonstoichiometric balanced complexes, which we have already shown to be present in real-world biochemical networks operating under flux constraints. Our results demonstrate that the effective deficiency of real-world biochemical networks is smaller than the classical deficiency, indicating the effects of reaction directionality and flux bounds on the variation of the complex formation rate vector at steady state.

## Introduction

Chemical reaction network theory (CRNT) has made seminal contributions to establishing necessary and/or sufficient conditions that a (bio)chemical network exhibits particular properties, such as: presence/absence of multiple steady state, stability of steady states, and robustness of steady-state concentrations^1^. The deficiency of a network is of central importance in this theory, and many steady-state concentration properties are guaranteed or precluded for networks of particular deficiency^2–4^. These results often hold for (bio)chemical networks whose reactions are endowed with (generalized) mass action kinetics. However, a notable feature of this theory is that it does not impose any physico-chemical constraints that real-world networks obey. As a result, the notion of deficiency is not concerned with the (ir)reversibility of the considered biochemical reactions that may arise in practice, and the extent to which this may affect the properties of the corresponding steady states the network supports.

In contrast to CRNT, which almost exclusively deals with concentration-based properties, the constraint-based modeling framework^5,6^, imposes physico-chemical constraints as lower and/or upper bounds on reaction rates (i.e. fluxes) in making predictions about macro-level phenotypes, such as growth or yield of a chemical product of interest. The constraint-based modeling framework has found numerous applications, largely due to its capacity to model large-scale networks by building on approaches for convex optimization.

A natural question then arises of how to integrate any imposed constraints on reaction fluxes into the notion of deficiency, and if the consideration of such constraints leads to smaller values such that one may expand the usability of the classical results from CRNT^2,3^. Here, we rely on the recently introduced classification of balanced complexes^7^, in particular the class of nonstoichiometric balanced complexes, to define the notion of effective deficiency of a network. Nonstoichiometric balanced complexes have been shown to arise as a combined result of several factors, including the algebraic and graphical structure of the network as well as operational bounds on reaction kinetics^7^. A remarkable feature of the effective deficiency is that, like that of the classical, so-called structural, it is defined and can be calculated for networks of arbitrary kinetics.

Our theoretical results show that decrease in the structural deficiency of a network, as quantified by the effective deficiency, is due to the presence of reactions whose fluxes are fixated at their lower bounds—i.e. they are robust irrespective of the environment. In other words, given the irreversibility and flux bound constraints, if the network contains nonstoichiometric balanced complexes, some reactions are guaranteed to exhibit absolute flux robustness. It is then not surprising that the effective deficiency captures the reduced capacity for variation of the complex formation rate at steady state. We conjecture that the effective deficiency, like the structural deficiency, can provide rich information about the properties of the steady states the network supports – a direction for future research in this area.

The paper is organized as follows: In Sect. “Background on CRNT”, we present the basics of chemical reaction network theory, following the terminology and notation introduced by Feinberg et al. such as linkage structure and deficiency, as well as notions from the constraint-based modeling literature, such as the flux space and its properties. Section “Mathematics of balanced complexes” builds on a previous work^7^, introduces balanced complexes, and explores the network properties that give rise to this phenomenon. Then, in Sect. “Properties of nonstoichiometric BCs”, we discuss an intriguing subclass of balanced complexes, the so-called nonstoichiometric balanced complexes, which arise as a result of constraints imposed on a network. We show that the presence of such complexes has some implications, not only for the structure of the network but also for its ability to admit steady states. In particular, by introducing the notion of phantom species, we show in Sect. “Nonstoichiometric BCs and the integration of phantom species” that networks with non-stoichiometric BCs are -in a sense- equivalent to other -modified- networks of smaller deficiencies. This paves the way for the concept of effective deficiency in Sect. “Effective deficiency”, which extends the classical notion of deficiency to networks under flux constraints. Judging from the similarity of the underlying capacity between the original network and modified networks with smaller deficiencies, we conjecture that the effective deficiency of the network may determine the exclusion of exotic steady state behavior. The presence of nonstoichiometric BCs has been illustrated by toy examples in Sect. “Some examples”, as well as by analyzing several genome-scale metabolic networks. For instance, application of our results on twelve real-world metabolic networks of species from all three kingdoms of life show that their effective deficiency is decreased by 8% on average in comparison to the structural deficiency. If our conjecture is correct, under further restrictive conditions, the smaller effective deficiency in comparison to the structural deficiency could be used in understating factors that preclude exotic dynamic behavior, as alluded in Sect. “Conclusion”, which concludes the paper.

## Background on CRNT

### Problem setup

A chemical reaction network (CRN) is defined by a set of $m$ *species*/*metabolites*,$\mathcal{S}={\left\{{S}_{i}\right\}}_{i=1}^{m}$, a set of $n$ *complexes*,$\mathcal{C}={\left\{{C}_{j}\right\}}_{j=1}^{n}$, each of which is a multiset of species ${C}_{j}\in {\mathbb{N}}^{\mathcal{S}}$, and a set of $r$ *reactions*,$\mathcal{R}={\left\{{R}_{q}\right\}}_{q=1}^{r}\subset \mathcal{C}\times \mathcal{C}$, which symbolize the potential conversion of complexes into each other in the network^2,3,8,9^.

We denote the standard basis in ${\mathbb{R}}^{n}$ by ${\left\{{\mathbf{e}}_{j}\right\}}_{j=1}^{n}$, where

$${\mathbf{e}}_{j}={\left[0 \cdots 0 1 0 \cdots 0\right]}^{\mathrm{T}} ,$$

is an $n-$ vector with a unit value at the $jth$ entry and zero entries elsewhere. Let us next assume some arbitrary ordering on the sets of species $({S}_{1},\cdots ,{S}_{m})$, complexes $\left({C}_{1},\cdots ,{C}_{n}\right)$, and reactions $({R}_{1},\cdots {R}_{r})$. Given this ordering, any complex ${C}_{j}\in \mathcal{C}$ can be associated with a vector ${\mathbf{e}}_{j}\in {\mathbb{R}}^{n}$ indexing its position in the ordered set, and at the same time with a unique vector ${\mathbf{y}}_{j}\in {\mathbb{R}}^{m}$, which represents its species content. This defines the following mapping

1 $${\mathbf{e}}_{j}\mapsto {\mathbf{y}}_{j}\in {\mathbb{R}}^{m} , 1\le j\le n .$$

Consequently, any given CRN is associated with a *stoichiometric map*, defined by the matrix $\mathbf{Y}=\left[{\mathbf{y}}_{1} \cdots {\mathbf{y}}_{n}\right]\in {\mathbb{R}}^{m\times n}$. Similarly, the ordering associates each reaction ${R}_{q} : {C}_{{p}{\prime}}\rightharpoonup {C}_{p}$, converting complex ${C}_{{p}{\prime}}$ to ${C}_{p}$, with a vector ${\mathbf{a}}_{q}={\mathbf{e}}_{p}-{\mathbf{e}}_{{p}{\prime}}\in {\mathbb{R}}^{n}$ that represents this conversion in the complex space, and a vector ${\mathbf{n}}_{q}={\mathbf{y}}_{p}-{\mathbf{y}}_{{p}{\prime}}\in {\mathbb{R}}^{m}$, which represents this conversion in the species space. Thus, the CRN is at the same time associated with the *complex—reaction incidence matrix* $\mathbf{A}=\left[{\mathbf{a}}_{1} \cdots {\mathbf{a}}_{r}\right]\in {\mathbb{R}}^{n\times r}$ and a species conversion matrix $\mathbf{N}=\left[{\mathbf{n}}_{1} \cdots {\mathbf{n}}_{r}\right]\in {\mathbb{R}}^{m\times r}$, known as *stoichiometry matrix*. It follows immediately from the definition of the stoichiometric map that $\mathbf{N}=\mathbf{Y}\mathbf{A} .$ The column span of $\mathbf{N}$ is called the *stoichiometric subspace*, the dimension of which,$s$, is termed *rank* of the CRN, that is,$s=\mathrm{rank}(\mathbf{N})$.

Assuming the reaction network is endowed with some kinetics, at any state of the system, denoted by the vector of concentrations $\mathbf{x}\in {\mathbb{R}}_{\ge 0}^{m}$, the dynamics is governed by the following ODE

2 $$\frac{d}{dt}\mathbf{x}=\mathbf{N}\mathbf{v}(\mathbf{x})=\mathbf{Y}\mathbf{A}\mathbf{v}(\mathbf{x}) ,$$

where $\mathbf{v}\left(\mathbf{x}\right)\in {\mathbb{R}}^{r}$ returns the reaction rates determined by system kinetics as a generally nonlinear function of the state vector $\mathbf{x}$.

### Blocked reactions and the steady state flux space

The vector $\mathbf{v}\in {\mathbb{R}}^{r}$, referred to as a *flux distribution* for the network $G=\left(\mathcal{S},\mathcal{C},\mathcal{R}\right)$, is said to be at steady state, if

3 $$\mathbf{N}\mathbf{v}=\mathbf{Y}\mathbf{A}\mathbf{v}=0\boldsymbol{ }.$$

In addition, the flux vector $\mathbf{v}$ is often assumed to be bounded by box constraints as follows

4 $${\mathbf{v}}_{\text{l}}\le \mathbf{v}\le {\mathbf{v}}_{\text{u}} .$$

where $\le$ operates element-wise, and ${\mathbf{v}}_{\text{l}},{\mathbf{v}}_{\text{u}}\in {\mathbb{R}}^{r}$ specify the individual lower- and upper bounds on flux through reactions, respectively. By convention,${\mathbf{v}}_{\text{u}}$ is strictly positive for all reactions $, {\mathbf{v}}_{\text{u}}>0$. Going forward, we assume a network denoted $G$ also encodes information about upper and lower bounds on reaction fluxes.

When no additional restriction is imposed on the network, a flux distribution $\mathbf{v}$ is called *feasible*, if it satisfies both constraints (3) and (4). The set of all feasible flux distributions is an intercept of some hyperplanes and halfspaces, which defines a convex polyhedron, referred to as the *steady state flux space*, denoted $\mathcal{F}\left(G\right)=\left\{\mathbf{v}\in {\mathbb{R}}^{r} | \mathbf{Y}\mathbf{A}\mathbf{v}=0, {\mathbf{v}}_{\text{l}}\preccurlyeq \mathbf{v}\preccurlyeq {\mathbf{v}}_{\text{u}}\right\}$.

The set of reversible reactions ${\mathcal{R}}^{\text{rev}}\subseteq \mathcal{R}$ is defined by negative entries of ${\mathbf{v}}_{\mathrm{l}}$, that is

$${\mathcal{R}}^{\text{rev}}=\left\{R\in \mathcal{R}|{v}_{{\text{l}},R}<0\right\} ,$$

where ${v}_{\mathrm{l},R}$ denotes the entry of ${\mathbf{v}}_{\mathrm{l}}$ corresponding to reaction $R$. The set of irreversible reactions ${\mathcal{R}}^{\text{irr}}\subseteq \mathcal{R}$ is then defined as the complement of ${\mathcal{R}}^{\text{rev}}$, that is,${\mathcal{R}}^{\text{irr}}=\mathcal{R}\setminus {\mathcal{R}}^{\text{rev}}$. Hence, irreversible reactions are associated with ${v}_{{\text{l}},R}\ge 0$. By contrast to irreversible reactions, a reversible reaction ${R}_{q} :{C}_{{p}{\prime}}\rightleftharpoons {C}_{p}$ portrays the mutual conversion of complexes ${C}_{{p}{\prime}}$ and ${C}_{p}$.

Similarly, let ${\mathcal{R}}^{\text{zl}}$ and ${\mathcal{R}}^{\text{nl}}$ denote the sets of reactions with zero and nonzero lower bounds, respectively. It follows from the definition that,${\mathcal{R}}^{\text{zl}}\cap {\mathcal{R}}^{\text{nl}}=\varnothing ; {\mathcal{R}}^{\text{zl}}\cup {\mathcal{R}}^{\text{nl}}=\mathcal{R};$ and ${\mathcal{R}}^{\text{zl}}\subseteq {\mathcal{R}}^{\text{irr}}$. Given the arbitrariness of indexing, one can always order the reactions in $\mathcal{R}$ such that elements of ${\mathcal{R}}^{\text{zl}}$ and ${\mathcal{R}}^{\text{irr}}$ precede those of ${\mathcal{R}}^{\text{nl}}$ and ${\mathcal{R}}^{\text{rev}}$, respectively. As a result, the incidence matrix $\mathbf{A}$ can be block-partitioned as either $\mathbf{A}=\left[{\mathbf{A}}^{\text{irr}}{\mathbf{A}}^{\text{rev}}\right]$ or $\mathbf{A}=\left[{\mathbf{A}}^{\text{zl}}{\mathbf{A}}^{\text{nl}}\right]$.

The network is said to be operating under a *canonical flux regime*, if ${\mathcal{R}}^{\text{zl}}={\mathcal{R}}^{\text{irr}}$. It is said to be operating under an *unbounded flux regime*, if ${\mathbf{v}}_{\text{u}}=\boldsymbol{\infty }$ and ${v}_{{\text{l}},R}=-\infty , \forall R\in {\mathcal{R}}^{\text{rev}}$. Under a canonical and unbounded flux regime, the set of feasible flux distributions forms a polyhedral convex cone, referred to as the *steady state flux cone*. The flux regime is called *bounded*, if it is not unbounded.

For any reaction $R\in \mathcal{R}$, we say $R$ is a *blocked reaction* in $G$, if for all flux distributions $\mathbf{v}\in \mathcal{F}\left(G\right)$, the flux through $R$ is zero, namely $, {v}_{R}\equiv 0$. This is a recurring phenomenon in metabolic networks, in particular when restrictive flux bounds and/or optimization of particular objectives are imposed. As long as we only concern ourselves with steady state analysis, all blocked reactions can be safely removed from the network.

More generally, we say a reaction $R\in \mathcal{R}$ is *fixated* (at some flux value $f$), if for all flux distributions $\mathbf{v}\in \mathcal{F}\left(G\right)$, the flux going through $R$ is unchanged, namely, ${v}_{R}=f, \forall \mathbf{v}\in \mathcal{F}\left(G\right)$. Obviously, the set of blocked reactions is contained in the set of fixated reactions.

### Linkage structure and deficiency

Two complexes $C,{C}{\prime}\in \mathcal{C}$ are said to be *directly linked*, if $\left(C,{C}{\prime}\right)\in \mathcal{R}$ or $\left({C}{\prime},C\right)\in \mathcal{R}$. Two complexes $C,{C}{\prime}\in \mathcal{C}$ are *linked*, denoted $C\sim {C}{\prime}$, if there exist a sequence of complexes $(C={C}_{{j}_{0}},{C}_{{j}_{1}},\cdots ,{C}_{{j}_{\kappa }}={C}{\prime})$, each of which is directly linked to the immediate preceding and succeeding elements. The equivalence relation $\sim$ partitions $\mathcal{C}$ into a family of $\ell$ equivalence classes ${\left\{{L}_{l}\right\}}_{l=1}^{\ell}$, called the *linkage classes* of the network.

For any two complexes $C,{C}{\prime}\in \mathcal{C}$, we say $C$ *directly converts to* $C{\prime}$, denoted $C\to {C}{\prime}$, if $\left(C,{C}{\prime}\right)\in \mathcal{R}$ or $\left({C}{\prime},C\right)\in {\mathcal{R}}^{\text{rev}}$. We say $C$ *converts to* $C{\prime}$, denoted $C\Rightarrow {C}{\prime}$, if there exist a sequence of complexes $(C={C}_{{j}_{0}}\to {C}_{{j}_{1}}\to \cdots \to {C}_{{j}_{\kappa }}={C}{\prime})$.$C$ and $C{\prime}$ are *strongly linked*, denoted $C\approx C{\prime}$, if $C\Rightarrow {C}{\prime}$ and ${C}{\prime}\Rightarrow C$. The equivalence relation $\approx$ partitions $\mathcal{C}$ into a family of ${\ell}_{\mathrm{s}}$ equivalence classes ${\left\{{\Lambda }_{l}\right\}}_{l=1}^{{\ell}_{\mathrm{s}}}$, called the *strong linkage classes* of the network.

A *terminal strong linkage class* is a strong linkage class $\Lambda$, no complex of which converts to any complex in $\mathcal{C}\backslash\Lambda$. We denote the number of terminal strong linkage classes by $\mathcal{t}$. It is trivial to show that, in general,${\ell}_{\mathrm{s}}\ge \mathcal{t}\ge \ell$. A reaction network is said to be *weakly reversible*, If $C\sim {C}{\prime}$ implies $C\approx C{\prime}$, that is, any two linked complexes convert to each other. In graph-theoretic terms, it means each component of the CRN graph—defined by complexes as vertices and reactions as directed edges – is strongly connected. For weakly reversible networks, every linkage class is a terminal strong linkage class, hence ${\ell}_{\mathrm{s}}=\mathcal{t}=\ell$.

The *deficiency* of a reaction network is the nonnegative integer, denoted $\delta$, defined by^2^

5 $$\delta =n-\ell-s .$$

Alternatively, it can be defined as^10,11^

6 $$\delta =\mathrm{dim}\left(\mathrm{ker}\left(\mathbf{Y}\right)\cap \mathrm{im}\left(\mathbf{A}\right)\right) .$$

As Eq. (6) demonstrates, the deficiency of a network has to do with the image of stoichiometric flux modes under linear mapping $\mathbf{A}$. In this light, for any network $G$, we define the *deficiency space* $\mathcal{D}\left(G\right)$ as the set of all feasible complex formation rate vectors, given as follows

$$\mathcal{D}\left(G\right)=\left\{\mathbf{a}\in {\mathbb{R}}^{n} | \mathbf{a}=\mathbf{A}\mathbf{v}, \mathbf{v}\in \mathcal{F}\left(G\right)\right\} .$$

Any nonzero vector $\mathbf{a}\in \mathcal{D}\left(G\right)$ points at a nonzero complex conversion, i.e. net production or consumption of some complexes at steady state, which is unobserved on the species level, because all species concentrations are constant. Thus, one may interpret the dimension of the deficiency space as a measure of the capacity of the network to permit unobserved complex conversions at steady state. This is why the notion of deficiency stands at the center of several seminal results in CRNT, which determine whether special classes of networks may exhibit multistationarity and/or exotic dynamical behavior^3^.

Given the definition of $\mathbf{A}$, it is not surprising that the linkage structure of the network is closely interlinked with the left nullspace of $\mathbf{A}$. Let $G$ be a closed network comprising $\ell$ linkage classes ${\left\{{L}_{l}\right\}}_{l=1}^{\ell}$. An obvious choice of a basis for the left nullspace of $\mathbf{A}$, namely $\mathrm{ker}{\mathbf{A}}^{\mathrm{T}}$, is the set ${\left\{{\mathbf{u}}^{\left(l\right)}\right\}}_{l=1}^{\ell}$, where ${\mathbf{u}}^{\left(l\right)}={\sum }_{j : {C}_{j}\in {L}_{l}} {\mathbf{e}}_{j} , 1\le l\le \ell$. Therefore, the column span of the *complex – linkage class incidence matrix*

7 $$\mathbf{U}=\left[{\mathbf{u}}^{\left(1\right)}\cdots {\mathbf{u}}^{\left(\ell\right)}\right]\in {\mathbb{R}}^{n\times \ell}\boldsymbol{ },$$

coincides with the left nullspace of $\mathbf{A}$. In a similar fashion, we can define the *complex – strong linkage class incidence matrix* as follows

8 $$\begin{array}{l}{\mathbf{U}}_{\text{s}}=\left[{\mathbf{u}}_{\text{s}}^{\left(1\right)}\cdots {\mathbf{u}}_{\text{s}}^{\left({\ell}_{\text{s}}\right)}\right]\in {\mathbb{R}}^{n\times {\ell}_{\text{s}}}\boldsymbol{ }\boldsymbol{ }\boldsymbol{ },\boldsymbol{ }\boldsymbol{ }\boldsymbol{ }\\ \boldsymbol{ }\boldsymbol{ }\boldsymbol{ }\boldsymbol{ }\boldsymbol{ }\boldsymbol{ }\boldsymbol{ }\boldsymbol{ }\boldsymbol{ }\boldsymbol{ }\boldsymbol{ }\boldsymbol{ }{\mathbf{u}}_{\text{s}}^{\left(l\right)}={\sum }_{j : {C}_{j}\in {\Lambda }_{l}} {\mathbf{e}}_{j} , 1\le l\le {\ell}_{\text{s}} .\end{array}$$

Since every linkage class is a disjoint union of some strong linkage classes, it follows that for any arbitrary network, ${\text{im}}\left(\mathbf{U}\right)\subseteq {\text{im}}\left({\mathbf{U}}_{\text{s}}\right)$, with equality holding only for weakly reversible networks.

## Mathematics of balanced complexes

Let us next give a formal definition of a balanced complex. For a given matrix $\mathbf{X}$, let ${\mathbf{X}}^{:i}$ and ${\mathbf{X}}^{j:}$ denote the $ith$ column and jth row of $\mathbf{X}$, respectively. A complex ${C}_{j}\in \mathcal{C}$ is referred to as a *balanced complex* (BC) for network $G$, if ${\mathbf{A}}^{j:}\mathbf{v}\equiv 0$ for all $\mathbf{v}\in \mathcal{F}\left(G\right)$. Given the properties of the incidence matrix $\mathbf{A}$, this definition complies with the notion that the algebraic sum of fluxes entering complex ${C}_{j}$ must be equal to the sum of fluxes leaving it for all feasible distributions $\mathbf{v}\in \mathcal{F}\left(G\right)$, which means this complex has a zero complex formation rate at all steady states.

The contents of this section build heavily upon earlier works in^12^ and^7^. We refer the readers interested in a more detailed discussion to these studies for more information.

### Factorizations of balanced complexes

As was shown in^7^, a complex ${C}_{j}\in \mathcal{C}$ is a BC, if and only if

9 $$\left\{\begin{array}{l}{\mathbf{e}}_{j}={\mathbf{Y}}^{\mathrm{T}}{{\varvec{\upzeta}}}_{1}+\mathbf{U}{{\varvec{\upxi}}}_{1}+{{\varvec{\uptheta}}}_{1}\\ {\mathbf{e}}_{j}={\mathbf{Y}}^{\mathrm{T}}{{\varvec{\upzeta}}}_{2}+\mathbf{U}{{\varvec{\upxi}}}_{2}-{{\varvec{\uptheta}}}_{2}\end{array},\right.$$

where variables ${{\varvec{\upzeta}}}_{1},\boldsymbol{ }{{\varvec{\upzeta}}}_{2}\in {\mathbb{R}}^{m} ,\boldsymbol{ }\boldsymbol{ }{{\varvec{\upxi}}}_{1},\boldsymbol{ }{{\varvec{\upxi}}}_{2}\in {\mathbb{R}}^{\ell}$ are free parameters, while the parameters ${{\varvec{\uptheta}}}_{1},\boldsymbol{ }{{\varvec{\uptheta}}}_{2}\in {\mathbb{R}}^{n}$ satisfy

$$\left\{\begin{array}{l}{\mathbf{A}}^{\mathrm{T}}{{\varvec{\uptheta}}}_{t}={{\varvec{\uplambda}}}_{{\text{l}}t}-{{\varvec{\uplambda}}}_{{\text{u}}t}\\ {{\mathbf{v}}_{\text{l}}}^{\mathrm{T}}{{\varvec{\uplambda}}}_{{\text{l}}t}-{{\mathbf{v}}_{\text{u}}}^{\mathrm{T}}{{\varvec{\uplambda}}}_{{\text{u}}t}=0\end{array} , t=\mathrm{1,2 }\right.,$$

for some nonnegative dual variables ${{\varvec{\uplambda}}}_{{\text{l}}1} , {{\varvec{\uplambda}}}_{{\text{u}}1} ,{{\varvec{\uplambda}}}_{{\text{l}}2} , {{\varvec{\uplambda}}}_{{\text{u}}2}\in {\mathbb{R}}_{\ge 0}^{r}$^7^.

The set of all balanced complexes in $G$, denoted $\mathcal{B}=\mathcal{B}\left(G\right)$, can then be defined as

10 $$\mathcal{B}=\left\{{C}_{j}\in \mathcal{C} \left|\begin{array}{l}\exists {{\varvec{\upzeta}}}_{1},\boldsymbol{ }{{\varvec{\upzeta}}}_{2}\in {\mathbb{R}}^{m}, {{\varvec{\uplambda}}}_{\mathrm{l}1}, {{\varvec{\uplambda}}}_{\mathrm{u}1},{{\varvec{\uplambda}}}_{\mathrm{l}2}, {{\varvec{\uplambda}}}_{\mathrm{u}2}\in {\mathbb{R}}_{\succcurlyeq 0}^{r} :\\ \left\{ \begin{array}{l}{\mathbf{A}}^{\mathrm{T}}{\mathbf{e}}_{j}={\mathbf{A}}^{\mathrm{T}}{\mathbf{Y}}^{\mathrm{T}}{{\varvec{\upzeta}}}_{1}+{{\varvec{\uplambda}}}_{{\text{l}}1}-{{\varvec{\uplambda}}}_{{\text{u}}1}\\ {\mathbf{A}}^{\mathrm{T}}{\mathbf{e}}_{j}={\mathbf{A}}^{\mathrm{T}}{\mathbf{Y}}^{\mathrm{T}}{{\varvec{\upzeta}}}_{2}+{{\varvec{\uplambda}}}_{{\text{u}}2}-{{\varvec{\uplambda}}}_{{\text{l}}2}\\ {{\mathbf{v}}_{\text{l}}}^{\mathrm{T}}{{\varvec{\uplambda}}}_{{\text{l}}t}-{{\mathbf{v}}_{\text{u}}}^{\mathrm{T}}{{\varvec{\uplambda}}}_{{\text{u}}t}=0 , t=\mathrm{1,2}\end{array} \right.\end{array}\right.\right\}.$$

A large subset of these balanced complexes can be characterized as follows

11 $${\mathcal{B}}_{1}=\left\{{C}_{j}\in \mathcal{C} \left|\begin{array}{l}\exists {{\varvec{\upzeta}}}_{1},\boldsymbol{ }{{\varvec{\upzeta}}}_{2}\in {\mathbb{R}}^{m}, {{\varvec{\upxi}}}_{1},\boldsymbol{ }{{\varvec{\upxi}}}_{2}\in {\mathbb{R}}^{\ell}, {{\varvec{\uptheta}}}_{1},\boldsymbol{ }{{\varvec{\uptheta}}}_{2}\in {\mathbb{R}}^{n} :\\ \left\{ \begin{array}{l}{\mathbf{e}}_{j}={\mathbf{Y}}^{\mathrm{T}}{{\varvec{\upzeta}}}_{1}+\mathbf{U}{{\varvec{\upxi}}}_{1}+{{\varvec{\uptheta}}}_{1}\\ {\mathbf{e}}_{j}={\mathbf{Y}}^{\mathrm{T}}{{\varvec{\upzeta}}}_{2}+\mathbf{U}{{\varvec{\upxi}}}_{2}-{{\varvec{\uptheta}}}_{2}\\ {{\mathbf{A}}^{\text{nl}}}^{\mathrm{T}} \left[{{\varvec{\uptheta}}}_{1}\boldsymbol{ }\boldsymbol{ }\boldsymbol{ }{{\varvec{\uptheta}}}_{2}\right]=0 \\ {{\mathbf{A}}^{\text{zl}}}^{\mathrm{T}} \left[{{\varvec{\uptheta}}}_{1}\boldsymbol{ }\boldsymbol{ }\boldsymbol{ }{{\varvec{\uptheta}}}_{2}\right]\succcurlyeq 0 \end{array} \right.\end{array}\right.\right\}.$$

In a canonical flux regime, the above two sets coincide^7^, i.e. $\mathcal{B}={\mathcal{B}}_{1}.$ Note that in that case,${\mathbf{A}}^{\text{nl}}={\mathbf{A}}^{\text{rev}}$ and ${\mathbf{A}}^{\text{zl}}={\mathbf{A}}^{\text{irr}}$.

Furthermore, in a canonical flux regime, if the network is void of any blocked reactions, the set of balanced complexes reduces to

12 $${\mathcal{B}}_{2}=\left\{{C}_{j}\in \mathcal{C} \left|\exists {\varvec{\upzeta}}\in {\mathbb{R}}^{m}, {\varvec{\upxi}}\in {\mathbb{R}}^{\ell} : {\mathbf{e}}_{j}={\mathbf{Y}}^{\mathrm{T}}{\varvec{\upzeta}}+\mathbf{U}{\varvec{\upxi}}\right.\right\},$$

a particular subset of which is given as follows

13 $${\mathcal{B}}_{3}=\left\{{C}_{j}\in \mathcal{C} \left|\exists {\varvec{\upzeta}}\in {\mathbb{R}}^{m} : {\mathbf{e}}_{j}={\mathbf{Y}}^{\mathrm{T}}{\varvec{\upzeta}}\right.\right\}.$$

The equalities in Eqs. (10), (11), (12) and (13), referred to as *factorizations*, explain the formation of a balanced complex ${C}_{j}\in \mathcal{C}$ as a combined effect of a number of underlying factors, namely, the stoichiometry ($\mathbf{Y}$), linkage structure ($\mathbf{U}$), irreversibility patters (${\mathcal{R}}^{\text{zl}}\subseteq {\mathcal{R}}^{\text{irr}}$) and flux bounds (${\mathbf{v}}_{\text{l}},{\mathbf{v}}_{\text{u}}$).

### Nonstoichiometric balanced complexes

It is worth noting that

14 $${\mathcal{B}}_{3}\subseteq {\mathcal{B}}_{2}\subseteq {\mathcal{B}}_{1}\subseteq \mathcal{B}.$$

A complex ${C}_{j}\in \mathcal{C}$ is called a *strictly stoichiometric BC*, if ${C}_{j}\in {\mathcal{B}}_{3}$; the equality in (13) is referred to as a *strictly stoichiometric factorization* for complex ${C}_{j}$. A complex ${C}_{j}\in \mathcal{C}$ is called a *stoichiometric BC*, if ${C}_{j}\in {\mathcal{B}}_{2}$; the equality in (12) is a *stoichiometric factorization* for complex ${C}_{j}$. By contrast, a complex ${C}_{j}\in \mathcal{C}$ is called a nonstoichiometric BC, if ${C}_{j}\in \mathcal{B}\backslash {\mathcal{B}}_{2}$.

The notion stoichiometric arises from the fact that due to the factorizations given in (12) and (13), the balancing property for such complexes relies heavily on the stoichiometric structure of the network, and not on other imposed constraints, such as: irreversibility and flux bounds. The notions are borrowed from^7^, to which we refer the interested reader for more details.

While all nonstoichiometric BCs have an implicit factorization of the form (10), we make a distinction between those which also have an explicit factorization of the form (11), and those which do not. A complex ${C}_{j}\in \mathcal{C}$ is called a type-I *nonstoichiometric BC*, if ${C}_{j}\in {\mathcal{B}}_{1}\backslash {\mathcal{B}}_{2}$, that is, it has a factorization of the form (11), but no stoichiometric factorization. A complex ${C}_{j}\in \mathcal{C}$ is called a type-II *nonstoichiometric BC*, if ${C}_{j}\in \mathcal{B}\backslash {\mathcal{B}}_{1}$, that is, it has a factorization of the form (10), but none of the form (11). A complex ${C}_{j}\in \mathcal{C}$ is *unbalanced*, if it has no factorization of the form (10), that is $, {C}_{j}\in \mathcal{C}\backslash B$.

The emergence of nonstoichiometric BCs is an intriguing phenomenon in (bio)chemical reaction networks. As will become clear in the next section, it reflects on some key structural properties of the reaction network. Furthermore, it also reflects on the reduced capacity of a network to permit variations of the complex formation rate vector at steady state.

## Properties of nonstoichiometric BCs

We hereby aim to seek conditions under which nonstoichiometric BCs may emerge in a network, and to see what implications their existence may have on structural properties of a reaction network. We begin by analyzing type-I nonstoichiometric BCs, which is the only viable type in canonical flux regimes. However, it shall be noted that they may also emerge in non-canonical flux regimes. The following statement was proven in^7^.

### Proposition 4.1

Given a network $G$, if the set ${\mathcal{B}}_{1}\setminus {\mathcal{B}}_{2}$ is nonempty, then $G$ contains at least two irreversible reactions which are blocked at steady state.

The proof follows from the fact that nonzero (positive) entries in vectors ${{\mathbf{A}}^{\text{zl}}}^{\mathrm{T}}{{\varvec{\uptheta}}}_{1}$ and ${{\mathbf{A}}^{\text{zl}}}^{\mathrm{T}}{{\varvec{\uptheta}}}_{2}$ must correspond to blocked reactions in ${\mathcal{R}}^{\text{zl}}$. Existence of ${C}_{j}\in {\mathcal{B}}_{1}\setminus {\mathcal{B}}_{2}$ requires that the vectors ${{\mathbf{A}}^{\text{zl}}}^{\mathrm{T}}{{\varvec{\uptheta}}}_{1}$ and ${{\mathbf{A}}^{\text{zl}}}^{\mathrm{T}}{{\varvec{\uptheta}}}_{2}$ are both nonzero and also not collinear. Proposition 4.1 shows how the presence of a nonstoichiometric BC reflects qualitatively on the steady state properties. In fact, the following statements show that it also contains information about the graphical structure of the CRN.

### Proposition 4.2

Let the complex ${C}_{j}\in {\mathcal{B}}_{1}$ have an explicit factorization of the form (11) with parameters ${{\varvec{\uptheta}}}_{1},\boldsymbol{ }{{\varvec{\uptheta}}}_{2}\in {\mathbb{R}}^{n}$. Then $, {{\varvec{\uptheta}}}_{1},\boldsymbol{ }{{\varvec{\uptheta}}}_{2}\in {\text{im}}\left({\mathbf{U}}_{\text{s}}\right)$.

For weakly reversible networks, ${\mathbf{U}}_{\text{s}}=\mathbf{U},$ and hence $, {\text{im}}\left({\mathbf{U}}_{\text{s}}\right)={\text{im}}\left(\mathbf{U}\right)$. Therefore, the parameter ${{\varvec{\uptheta}}}_{t}$ can be removed by merging its value with the term $\mathbf{U}{{\varvec{\upxi}}}_{t}$, which yields a stoichiometric factorization for each ${C}_{j}\in {\mathcal{B}}_{1}$. As a result, type-I nonstoichiometric BCs do not emerge in weakly reversible reaction networks.

### Proposition 4.3

Given a network $G$, if the set ${\mathcal{B}}_{1}\setminus {\mathcal{B}}_{2}$ is nonempty, then $G$ is not weakly reversible and ${\ell}_{\text{s}}\ge \ell+2$.

In particular, it can be shown that blocked irreversible reactions indexed by strictly positive entries of ${{\mathbf{A}}^{\text{zl}}}^{\mathrm{T}}{{\varvec{\uptheta}}}_{t}, t=\mathrm{1,2}$ are “bridge reactions” that connect distinct strong linkage classes of the network. In fact, removal of all such blocked reactions increases the number of linkage classes by at least two. Proposition 4.3 shows an interesting contrast to a well-established result in chemical reaction network theory, which states that (full) complex balancing can be obtained at a positive steady state, only if the network is weakly reversible [Proposition 16.5.7 in^1^].

Interestingly, once the blocked reactions predicted by Proposition 4.1 are removed, a number of strong linkage classes will be detached from the rest of the network and form separate linkage classes.

### Proposition 4.4

For a network $G$, let ${C}_{j}\in {\mathcal{B}}_{1}$. Let $\underline{G}$ be the network obtained by removing all blocked reactions in $G$. Then ${C}_{j}\in \underset{\_}{{\mathcal{B}}_{2}}$, that is $, {C}_{j}$ is a stoichiometric BC in $\underline{G}$. Furthermore $, \underset{\_}{\ell}\ge \ell+2$.

The following corollary follows immediately from Proposition 4.1.

### Corollary 4.5

Let $G$ be a network with all its blocked reactions have been removed; then we have ${\mathcal{B}}_{1}={\mathcal{B}}_{2}$ and $\mathcal{B}\setminus {\mathcal{B}}_{1}=\mathcal{B}\setminus {\mathcal{B}}_{2} ;$ that is, all nonstoichiometric BCs are of type II.

By contrast to type-I nonstoichiometric BCs, the type-II BCs do not necessarily require the network to contain blocked reactions at steady state; hence, they do not automatically follow from modified topological features of the network at steady state. However, a rather similar trend could be observed: For a type-II nonstoichiometric BC to exist, a number of reactions must be fixated at nonzero lower- or upper bounds.

### Proposition 4.6

Let $G$ be a network, all blocked reactions of which have been removed. Suppose $\mathcal{B}\setminus {\mathcal{B}}_{1}$ is nonempty. Then $G$ contains at least three reactions fixated at a corresponding nonzero lower- or upper bound, for all $\mathbf{v}\in \mathcal{F}\left(G\right)$.

One can actually come up with a slightly more informative statement.

### Proposition 4.7

For a network $G$, suppose $\mathcal{B}\setminus {\mathcal{B}}_{1}$ is nonempty. Then $G$ contains a reaction $R\in {\mathcal{R}}^{\text{irr}}\setminus {\mathcal{R}}^{\text{zl}}$ fixated at a positive lower bound. Moreover, there exists another reaction ${R}{\prime}\in \mathcal{R}$ fixated at a positive upper bound, or a reaction ${R}{\prime}\in {\mathcal{R}}^{\text{rev}}$ fixated at a negative lower bound.

Note that none of these results imposes any specific restrictions on the graphical structure of the network, unlike the type-I case. For type-II nonstoichiometric BCs, it is not the topological feature but the imposed flux constraints that set the stage for the emergence of additional balanced complexes. The next result follows immediately from Proposition 4.7.

### Corollary 4.8

Given a network $G$, for the set $\mathcal{B}\setminus {\mathcal{B}}_{1}$ to be nonempty,$G$ must operate in a bounded and non-canonical flux regime.

Conversely, suppose $G$ is a network operating under an unbounded and/or canonical flux regime, and it contains no blocked reactions. Then, $\mathcal{B}={\mathcal{B}}_{1}={\mathcal{B}}_{2}$.

## Nonstoichiometric BCs and the integration of phantom species

Balanced complexes have been shown to play a key role in the reduction of large-scale metabolic networks [see^12^ and references therein]. They shall be viewed as intrinsic properties of the network that shed light on its steady state characteristics regardless of the underlying kinetics governing the conversion of species. The nonstoichiometric BCs have an additional interesting facet to themselves: They carry extra information about the network structure, which is not contained in the steady state Eq. (3) or in the *linkage closedness condition* ${\mathbf{U}}^{\text{T}}\mathbf{A}\mathbf{v}=0$, but emerges as a joint product of the stoichiometry, graphical structure, and flux constraints.

In what follows, we introduce transformations on a given reaction network $G$, which encodes these extra pieces of information into the steady state equation of a modified network ${G}^{*}$, which has the same graphical but a different algebraic structure. This will enable us to exploit ${G}^{*}$ to make observations about steady-state characteristics of the original CRN $G$. Even though only nonstoichiometric BCs carry extra pieces of information to encode, we shall next begin by introducing these transformations in the more general case, i.e. for any arbitrary BC ${C}_{b}\in \mathcal{C}$.

### Insertion of phantom species

Let $G$ be a CRN and ${C}_{b}\in \mathcal{B}(G)$ be any balanced complex in $G$, i.e. ${{\mathbf{e}}_{b}}^{\text{T}}\mathbf{A}\mathbf{v}=0, \forall \mathbf{v}\in \mathcal{F}\left(G\right)$. Suppose we ‘amend’$G$ by inserting one molecule of some phantom species $\sigma$ into complex ${C}_{b}$. This yields the modified network ${G}^{*}=({\mathcal{S}}^{*},{\mathcal{C}}^{*},{\mathcal{R}}^{*})$, where ${\mathcal{S}}^{*}=\mathcal{S}\cup \left\{\sigma \right\} , {\mathcal{C}}^{*}=\left\{{\mathbf{y}}_{b}\oplus \sigma \right\}\cup \mathcal{C}\setminus \left\{{\mathbf{y}}_{b}\right\} , {\mathcal{R}}^{*}=\mathcal{R}$. Accordingly, the stoichiometric map and incidence matrix for ${G}^{*}$ are given by

$${\mathbf{Y}}^{*}=\left[\begin{array}{c}\mathbf{Y}\\ {{\mathbf{e}}_{b}}^{\text{T}}\end{array}\right] , {\mathbf{A}}^{\mathbf{*}}=\mathbf{A} .$$

We refer to this transformation as *injection of vector* ${\mathbf{e}}_{b}$ *into network* $G$. As this procedure is only applied to some balanced complex ${C}_{b}$ and it involves insertion of some new species (not already in the network), the following result is trivially obtained.

#### Lemma 5.1

Let $G$ be a network,${C}_{b}\in \mathcal{B}\left(G\right)$ and ${G}^{*}$ the modified network obtained by injection of ${\mathbf{e}}_{b}$ into $G$. Then $G$ and ${G}^{*}$ have identical steady state flux distributions, that is, $\mathcal{F}\left(G\right)=\mathcal{F}\left({G}^{*}\right)$. In particular, any balanced complex of one is a balanced complex of the other, $\mathcal{B}={\mathcal{B}}^{*}$. Moreover, we have the inclusion ${\mathcal{B}}_{2}\subseteq {\mathcal{B}}_{2}^{*}$, which turns to a strict inclusion, if ${C}_{b}\in \mathcal{B}\setminus {\mathcal{B}}_{2}$.

While $G$ and ${G}^{*}$ have identical balanced complexes, the stoichiometric nature of BCs may differ across the two networks. In particular, for the modified complex, we have ${C}_{b}\in {\mathcal{B}}_{3}^{*} ,$ regardless of its original categorization in $G$. For any chosen ${C}_{b}\in \mathcal{B}$ , this transformation preserves the steady state flux space, and the information carried by any such BC is hereby encoded into the steady state equation. Clearly, the number of nonstoichiometric BCs strictly decreases in this process, in case of ${C}_{b}\in \mathcal{B}\setminus {\mathcal{B}}_{2}$.

### Characteristics of the modified network

The next question to address is how the stoichiometric subspace develops under the introduced transformation.

#### Proposition 5.2

Let $G$ be a network,${C}_{b}\in \mathcal{B}\left(G\right)$ and ${G}^{*}$ the modified network obtained by injection of ${\mathbf{e}}_{b}$ into $G$. Let us denote the stoichiometry matrices of $G$ and ${G}^{*}$ by $\mathbf{N}$ and ${\mathbf{N}}^{*}$, respectively. Then,

15 $${\text{rank}}\left({\text{N}}^{*}\right)={\text{rank}}\left(\mathbf{N}\right)+1 ,$$

if and only if ${C}_{b}\in \mathcal{B}\backslash {\mathcal{B}}_{2}$. Moreover, ${\text{rank}}\left({\text{N}}^{*}\right)={\text{rank}}\left(\mathbf{N}\right) ,$ if and only if ${C}_{b}\in {\mathcal{B}}_{2}$.

It is only the injection of nonstoichiometric BCs that increases the rank of the stoichiometric subspace. This yields the following corollary.

#### Corollary 5.3

Let $G, {G}^{*}$ be as defined in Proposition 5.2, and ${C}_{b}\in \mathcal{B}\backslash {\mathcal{B}}_{2}\left(G\right)$. Then, $\mathrm{dimker}\left({\text{N}}^{*}\right)=\mathrm{dimker}\left(\mathbf{N}\right)-1 .$

On the face of it, this seems to contradict the statement of Lemma 5.1. However, the ambiguity is easily sorted out, because not every vector in $\mathrm{ker}\left(\mathbf{N}\right)$ lies in $\mathcal{F}\left(G\right)$, simply due to irreversibility patterns and flux bounds. The apparent difference is only due to the fact that the extra piece of information carried by nonstoichiometric BC ${C}_{b}$ is now encoded in the steady state equation ${\mathbf{N}}^{\mathbf{*}}\mathbf{v}=0$.

The following result follows readily from the definition of deficiency:

#### Proposition 5.4

Let $G$ be a network,${C}_{b}\in \mathcal{B}\left(G\right)$ and ${G}^{*}$ the modified network obtained by injection of ${\mathbf{e}}_{b}$ into $G$. Let us denote the deficiency of $G$ and ${G}^{*}$ by $\delta$ and ${\delta }^{*}$, respectively. Then.

16 $${\delta }^{*}=\delta -1 ,$$

if and only if ${C}_{b}\in \mathcal{B}\setminus {\mathcal{B}}_{2}$. Moreover, ${\delta }^{*}=\delta ,$ if and only if ${C}_{b}\in {\mathcal{B}}_{2}$.

The deficiency is often characterized as a measure of how tightly the complex formation rate vector, i.e. the image of the steady state flux space under mapping $\mathbf{A}$, is constrained at steady state^1^, that is, the algebraic dimension of the deficiency space. Let us next focus on cases where ${C}_{b}$ is a nonstoichiometric BC. Given that $G$ and ${G}^{*}$ have the exact same flux distributions and the exact same incidence matrix, the contrast in their deficiency values is intriguing.

It is worth noting that the deficiency, as defined in (5) or (6), completely neglects of the fine details of the directionality of reactions, or the active bounds on flux through reactions. *The reaction arrows exert their influence only to the extent that they serve to partition the complexes into linkage classes*^1^. For the rather abstract notion of unconstrained networks, it is trivial to show that the deficiency value coincides with the dimension of the deficiency space, that is

$$\mathrm{dim}\mathcal{D}\left(G\right)=\mathrm{dim}\left(\mathrm{ker}\left(\mathbf{Y}\right)\cap \mathrm{im}\left(\mathbf{A}\right)\right)=\delta .$$

However, in more practical settings, e.g. the constrained framework presented in flux balance analysis of metabolic networks, the deficiency –as defined– should be understood only as an upper bound on the dimension of the deficiency space, that is, the following more general relation holds

$$\mathrm{dim}\mathcal{D}\left(G\right)\le \mathrm{dim}\left(\mathrm{ker}\left(\mathbf{Y}\right)\cap \mathrm{im}\left(\mathbf{A}\right)\right)=\delta .$$

In the case of $G$ and ${G}^{*}$, the two networks form identical complex formation rate vectors at steady state, that is, have identical deficiency spaces. However, the nominal deficiency of $G$,$\delta ,$ is larger than that of ${G}^{*}$, i.e.${\delta }^{*}=\delta -1$. This suggests that, due to abovementioned factors, the network $G$ is in fact more constrained at steady state than is reflected by $\delta =\mathrm{ker}\left(\mathbf{Y}\right)\cap \mathrm{im}\left(\mathbf{A}\right)$. Those factors cause the deficiency of $G$ to be *effectively* less than $\delta$, and no larger than ${\delta }^{*}$.

## Effective deficiency

Let $G$ be a network and $\mathcal{B}\setminus {\mathcal{B}}_{2}={\left\{{C}_{{b}_{j}}\right\}}_{j=1}^{\left|\mathcal{B}\setminus {\mathcal{B}}_{2}\right|}$ be the set of nonstoichiometric BCs for $G$. Let ${G}_{1}$ be the network obtained by injection of vector ${\mathbf{e}}_{{b}_{1}}$ into $G$. Given the discussion in Section “Insertion of phantom species”$, {\delta }_{1}=\delta -1$. Moreover, the equality $\mathcal{B}\left(G\right)=\mathcal{B}\left({G}_{1}\right)$ and strict inclusion ${\mathcal{B}}_{2}\left(G\right)\subset {\mathcal{B}}_{2}\left({G}_{1}\right)$ hold. Even though the remaining elements in $\mathcal{B}\setminus {\mathcal{B}}_{2}$, i.e. ${ \left\{{C}_{{b}_{j}}\right\}}_{j=2}^{\left|\mathcal{B}\setminus {\mathcal{B}}_{2}\right|}$ are balanced complexes of ${G}_{1}$, not all of them are necessarily a nonstoichiometric BC for ${G}_{1}$. However, any nonstoichiometric BC of ${G}_{1}$ must lie in ${\left\{{C}_{{b}_{j}}\right\}}_{j=2}^{\left|\mathcal{B}\setminus {\mathcal{B}}_{2}\right|}$.

Now suppose some member of ${\left\{{C}_{{b}_{j}}\right\}}_{j=2}^{\left|\mathcal{B}\setminus {\mathcal{B}}_{2}\right|}$ is a nonstoichiometric BC for ${G}_{1}$. Without loss of generality, let it be labelled ${C}_{{b}_{2}}$. Let ${G}_{2}$ be the network obtained by injection of vector ${\mathbf{e}}_{{b}_{2}}$ into ${G}_{1}$. It follows that ${\delta }_{2}={\delta }_{1}-1$. We then proceed further by searching the set ${\left\{{C}_{{b}_{j}}\right\}}_{j=3}^{\left|\mathcal{B}\setminus {\mathcal{B}}_{2}\right|}$ for any complex that may lie in $\mathcal{B}\setminus {\mathcal{B}}_{2}\left({G}_{2}\right)$. If it exists, we construct ${G}_{3}$ by injection of the corresponding vector, and so on.

As long as the most recent network in the sequence $G\to {G}_{1}\to {G}_{2}\to \cdots$ has any nonstoichiometric BC, one can basically repeat this process of inserting distinct phantom species and obtain the next modified network in the sequence. After repeating this process consecutively $d$ times, for some $d$, we will obtain ${\mathcal{B}}_{2}\left({G}_{d}\right)=\mathcal{B}$; that is, there remains no nonstoichiometric BC left to carry on with the process.

Our first observation is that, by virtue of Lemma 5.1, all networks $G, {G}_{1},\cdots , {G}_{d}$ have identical steady state flux distributions. Since they all have the same incidence matrix, they also share the same deficiency space, i.e. the steady state variations of the complex formation rate vector is also identical across all such networks. However, by virtue of Proposition 5.4$, {\delta }_{d}={\delta }_{d-1}-1={\delta }_{d-2}-2=\cdots =\delta -d$. It follows that, due to constraints on the flux space imposed by irreversibility patterns and flux bounds, the deficiency of network $G$ is effectively no larger than $\delta -d$.

In order to be able to define the notion of *effective deficiency* unambiguously, the question of uniqueness needs to be addressed: Had one chosen a different order for the sequence of nonstoichiometric BCs to inject and construct $G\to {G}_{1}\to {G}_{2}\to \cdots$, would one have still obtained the same value for $d$? The next statement addresses this question.

### Theorem 6.1

The maximum length of the sequence $G\to {G}_{1}\to \cdots \to {G}_{d}$, that is,$d$ is independent of the choice and order of nonstoichiometric BCs used to construct it.

Theorem 6.1 paves the way for the definition of effective deficiency for networks under irreversibility constraints and flux bounds.

### Definition

Let $G$ be a network and $\mathcal{B}\setminus {\mathcal{B}}_{2}$ the set of nonstoichiometric BCs for $G$. Let ${G}_{1},\cdots ,{G}_{d}$ be any arbitrary sequence of modified networks constructed iteratively by means of injecting nonstoichiometric BCs, and suppose ${\mathcal{B}}_{2}\left({G}_{d}\right)=\mathcal{B}$. The effective deficiency of $G$, denoted ${\delta }^{\text{eff}}$, is defined as follows.

17 $${\delta }^{\text{eff}}=\delta -d .$$

Note that $0\le d\le \left|\mathcal{B}\setminus {\mathcal{B}}_{2}\right|$. It follows that $\delta -\left|\mathcal{B}\setminus {\mathcal{B}}_{2}\right|\le {\delta }^{\text{eff}}\le \delta$. The following result facilitates the computation of effective deficiency, without having to explicitly construct any sequence of modified networks.

### Theorem 6.2

Suppose $\mathcal{B}\setminus {\mathcal{B}}_{2}={\left\{{C}_{{b}_{j}}\right\}}_{j=1}^{\left|\mathcal{B}\setminus {\mathcal{B}}_{2}\right|}$ is the set of nonstoichiometric BCs for $G$. Let us construct the matrix ${\mathbf{E}}_{\mathcal{B}\setminus {\mathcal{B}}_{2}}$ as ${\mathbf{E}}_{\mathcal{B}\setminus {\mathcal{B}}_{2}}=\left[{\mathbf{e}}_{{b}_{1}} {\mathbf{e}}_{{b}_{2}} \cdots {\mathbf{e}}_{{b}_{\left|\mathcal{B}\setminus {\mathcal{B}}_{2}\right|}}\right]$. The effective deficiency of $G$ is equal to.

18 $${\delta }^{\text{eff}}=\delta +{\text{rank}}\left(\mathbf{N}\right)-{\text{rank}}\left(\left[\begin{array}{c}\mathbf{N}\\ {{\mathbf{E}}_{\mathcal{B}\setminus {\mathcal{B}}_{2}}}^{\text{T}}\mathbf{A}\end{array}\right]\right).$$

One can seamlessly replace the set $\mathcal{B}\setminus {\mathcal{B}}_{2}$ in Proposition 6.2 with the set of all BCs $\mathcal{B}$ (and replace ${\mathbf{E}}_{\mathcal{B}\setminus {\mathcal{B}}_{2}}$ by ${\mathbf{E}}_{\mathcal{B}}$, which contains all balancing vectors as columns). Nevertheless, the exact same equality will still hold:

19 $${\delta }^{\text{eff}}=\delta +{\text{rank}}\left(\mathbf{N}\right)-{\text{rank}}\left(\left[\begin{array}{c}\mathbf{N}\\ {{\mathbf{E}}_{\mathcal{B}}}^{\text{T}}\mathbf{A}\end{array}\right]\right) .$$

However, the same value of ${\delta }^{\text{eff}}$ would be obtained at the expense of a higher computational effort. This is due to the fact that injection of stoichiometric BCs has neither an impact on the deficiency of the network nor on its flux distributions, simply because it incorporates no additional information into the stoichiometric structure.

We remark that, by the virtue of Eqs. (18) or (19), once the balanced complexes have been identified, obtaining the effective deficiency reduces to rank computation, which can be conducted by performing Gaussian elimination to obtain the row echelon form. Hence, the computation of effective deficiency has a time complexity of $\mathcal{O}\left({n}^{3}\right)$, at most.

In more technical terms, it can be shown that the effective deficiency has to do with the codimension of ${\text{im}}\left({\mathbf{Y}}^{\text{T}}\right)+{\text{im}}\left(\mathbf{U}\right)$, as stated in the following proposition.

### Proposition 6.3

Suppose $\mathcal{B}={\left\{{C}_{{b}_{j}}\right\}}_{j=1}^{\left|\mathcal{B}\right|}$ be the set of BCs for $G$, and ${\mathbf{E}}_{\mathcal{B}}$ defined as ${\mathbf{E}}_{\mathcal{B}}=\left[{\mathbf{e}}_{{b}_{1}} {\mathbf{e}}_{{b}_{2}} \cdots {\mathbf{e}}_{{b}_{\left|\mathcal{B}\right|}}\right]$. Then, the drop in deficiency,$d$, is the codimension of ${\text{im}}\left({\mathbf{Y}}^{\text{T}}\right)+{\text{im}}\left(\mathbf{U}\right)$ in ${\text{im}}\left({\mathbf{Y}}^{\text{T}}\right)+{\text{im}}\left(\mathbf{U}\right)+{\text{im}}\left({\mathbf{E}}_{\mathcal{B}}\right)$, that is.

20 $$d=\mathrm{dim}\left({\text{im}}\left({\mathbf{Y}}^{\text{T}}\right)+{\text{im}}\left(\mathbf{U}\right)+{\text{im}}\left({\mathbf{E}}_{\mathcal{B}}\right)/{\text{im}}\left({\mathbf{Y}}^{\text{T}}\right)+{\text{im}}\left(\mathbf{U}\right)\right) ,$$

where $V/W$ denotes the quotient space of $V$ by $W$.

Considering that the effective deficiency reflects the true dimension of the deficiency space in a network under functional and operational constraints, it is conceivable that it is in fact the effective deficiency, and not the nominal value of (structural) deficiency, that determines the steady state characteristics of a metabolic network, e.g. allowing unique equilibrium and excluding exotic dynamic behavior. We present this intuition as the following conjecture.

### Conjecture 6.4

Let $G$ be a network of arbitrary deficiency, potentially larger than one. Suppose the effective deficiency of $G$ satisfies either the conditions of Deficiency Zero Theorem or those of Deficiency One Theorem. Then, the results of the corresponding theorem, such as uniqueness of the equilibrium also apply to G.

While we do not prove this statement here, we would like to emphasize that all the results presented here point at this property. In particular, we have shown that there is a one-to-one correspondence between the flux space of $G$ and that of the modified networks obtained by insertion of the phantom species, which reflects itself in equivalent deficiency spaces. It seems only logical that if a modified network does not exhibit exotic steady state behavior, the same property should hold for $G$.

If the conjecture holds, it can have interesting implications even for large metabolic networks. Consider a metabolic network whose linkage classes are (i) independent subnetworks, and (ii) each of effective deficiency zero or one. Such a network then, by this conjecture, cannot admit more than a single steady state within any positive stoichiometric compatibility class, regardless of the values of the rate constants^1,2^.

## Some examples

In Section “Effective deficiency”, we introduced the notion of effective deficiency, and established its relation to the existence of nonstoichiometric BCs in the network. In the following, we illustrate this phenomenon via a number of examples. Here, we present toy networks, the design of which does not necessarily reflect real-world chemical or metabolic pathways, but is actually tailored for the purpose of illustration. On that account, the unrealistic nature of these toy networks shall not be viewed critically, as the goal is to simply show a couple of nonstoichiometric BCs and their consequences within a small and representable setting.

As far as real-world applications are concerned, the presence of nonstoichiometric BCs across a wide range of metabolic models has already been confirmed in a recent study^7^, which implies the exact same consequences for the effective deficiency of those metabolic networks.

### Toy network I: a type-I nonstoichiometric BC

**Figure 1:**
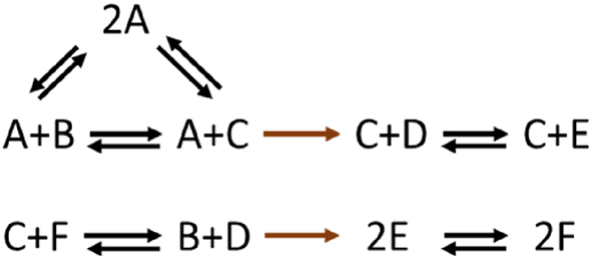
A type-I nonstoichiometric BC in a toy network. The basic conversion diagram depicts a network with $m=6$ species, $n=9$ complexes, and $r=8$ reactions. The network does not contain any stoichiometric BC. However, it contains exactly one type-I nonstoichiometric BC (complex $2A$). In line with the prediction of Proposition 4.1, the network also contains two blocked irreversible reactions (highlighted in brown).

Let us consider the toy network in Fig. 1.

The basic conversion diagram presented in Fig. 1 (including the reactions highlighted in brown) portrays a network operating in a canonical flux regime with $n=9$ complexes,$\ell=2$ linkage classes and of rank $s=5$. As a result, the network is of deficiency $\delta =n-\ell-s=2$. It contains no stoichiometric BCs. However, it can be shown that the complex ($2A$) has a nonstoichiometric factorization of the form Eq. (11), hence, it is a type-I nonstoichiometric BC. The detailed parameter values for the factorization are presented in the Supplementary.

As predicted by Proposition 4.1, the presence of a type-I nonstoichiometric BC is accompanied by having two irreversible reactions blocked at steady state, both of which are highlighted in brown. Moreover, $(2A)$ is the only balanced complex, hence, $d$ is bound from below and above to be exactly one. Therefore, while the network’s nominal deficiency is two, it follows from the above results that the network is of effective deficiency ${\delta }^{\text{eff}}=\delta -d=1$.

Let us next consider the reduced network obtained by removing all blocked reactions, but including all reactions shown in black. The reduced network will contain no nonstoichiometric BCs, since the balanced complex $\left(2A\right)$ has turned into a stoichiometric BC in this network. It has ${n}{\prime}=9$ complexes,${\ell}{\prime}=4$ linkage classes and rank ${s}{\prime}=4$. It follows that the reduced network is of deficiency ${\delta }{\prime}={n}{\prime}-{\ell}{\prime}-{s}{\prime}=1$. This is in accord with the value calculated for the effective deficiency of the original network.

### Toy network II: a type-II nonstoichiometric BC

**Figure 2:**
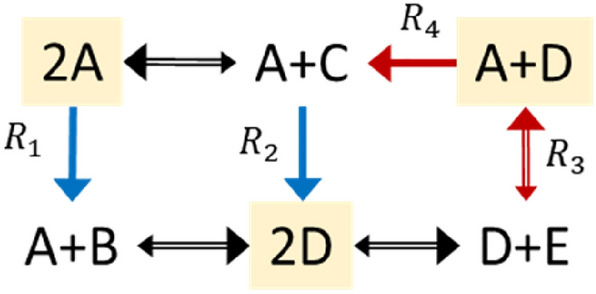
Type-II nonstoichiometric BCs in a toy network. The conversion diagram portrays a network with $m=5$ species, $n=6$ complexes, and $r=7$ reactions. The network operates in a non-canonical and bounded flux regime, where the fluxes of irreversible reactions ${R}_{1}$ and ${R}_{2}$ have strictly positive lower bounds (${v}_{{\text{l}},1}={v}_{{\text{l}},2}=100$), while the fluxes of reactions ${R}_{3}$ and ${R}_{4}$ have finite upper bounds (${v}_{{\text{u}},3}={v}_{{\text{u}},4}=200$). The network contains three type-II nonstoichiometric BCs, highlighted in yellow. The rest of complexes are stoichiometric BCs.

Next, let us consider the conversion diagram shown in Fig. 2.

For the reversible reactions, the larger arrow size depict the direction of the flux associated with a positive sign. In line with the prediction of Propositions 4.6 and 4.7, the network contains a number of reactions fixated at corresponding lower- and upper bounds, shown in blue and red, respectively.

The toy example in Fig. 2 portrays a network operating in a non-canonical and bounded flux regime. The three complexes $\left(A+B\right)$, $\left(A+C\right)$ and $\left(D+E\right)$ are strictly stoichiometric BCs, due to the fact that species $B$,$C$, and $E$ do not appear elsewhere in the toy network. The network has $n=6$ complexes,$\ell=1$ linkage class and rank $s=4$. Hence, the nominal deficiency of this network is $\delta =n-\ell-s=6-1-4=1$.

The other three complexes, that is,$\left(2A\right), \left(A+D\right)$ and $\left(2D\right)$ are type-II nonstoichiometric BCs. One can basically associate each with a nonstoichiometric factorization of the form (10) (details are available in the Supplementary). The existence of a nonstoichiometric BC implies that the effective deficiency ${\delta }^{\text{eff}}$ of this network must be strictly smaller than its nominal deficiency. Coupled with the knowledge that the deficiency only takes nonnegative values, it follows that ${\delta }^{\text{eff}}=0$. In other words, this is effectively a deficiency zero network, and thereby it is complex-balanced.

In line with the statements of Propositions 4.6 and 4.7, the network contains a number of reactions fixated at their lower- and upper bounds, shown in blue and red, respectively. It is worth noting that the formation of nonstoichiometric BCs in this network rely on the irreversibility of ${R}_{1}$ and ${R}_{2}$, the non-canonical flux regime imposed by strictly positive lower bounds on fluxes of of ${R}_{1}$ and ${R}_{2}$, as well as the unbounded flux regime imposed by finite upper bounds on fluxes of of ${R}_{3}$ and ${R}_{4}$. These *imposed constraints* force the network to perform like a deficiency-zero network and hence be complex-balanced, despite the fact that its underlying structure had the capacity to perform like a network of higher deficiency.

### Effective deficiency of large-scale metabolic networks

**Figure 3:**
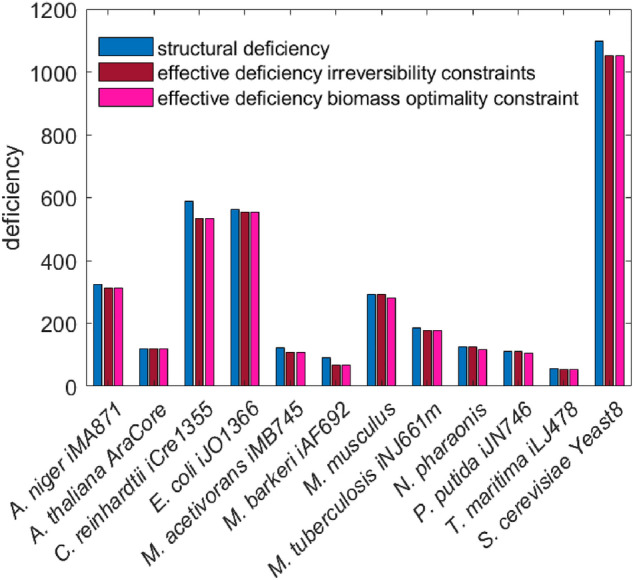
Structural and effective deficiency of real-world metabolic networks. Balanced complexes are identified in networks of twelve species from all kingdoms of life under two different scenarios: (i) imposing reaction irreversibility constraints, and (ii) considering in addition specific growth rate optimality. The effective deficiency is smaller than the structural deficiency when the additional constraint on optimality of specific growth rate is imposed for all networks.

We use twelve genome-scale metabolic networks^13–24^ from all kingdoms of life obtained from Küken et al.^12^ to investigate deficiency in networks with two sets of constraints (i) imposing reaction irreversibility constraints as specified in the original model reconstruction, and (ii) imposed optimality of specific growth rate in addition to reaction reversibility constraints. Note that blocked reactions are removed from the networks before BC detection and, therefore, the networks do not contain type-I nonstoichiometric BCs. In other words, all type-I nonstoichiometric BCs are transformed into stoichiometric BCs as a result of these removals (see Proposition 4.4). In this setting, we compare the structural deficiency and effective deficiencies obtained under the two different sets of constraints. The structural deficiency ranges from 57 for *T. maritima* to 1097 for *S. cerevisiae* (Fig. 3). We find that the effective deficiency obtained from scenario (i) is the same with the structural deficiency for the networks of *A. thaliana*, *M. musculus*, *N. pharaonis*, and *P. putida.* For the remaining eight networks, we find a reduction of the effective deficiency in comparison to the structural deficiency, ranging from 1.8% in *E. coli* and 35.3% in *M. barkeri* (Fig. 3). Considering the additional constraint on the specific growth rate, fixed at its optimum, in scenario (ii) we observe the effective deficiency to be smaller than the structural deficiency in all networks, with the smallest decrease in networks of *A. thaliana* (0.8%), *E. coli* (1.8%), *M. musculus* (3.6%) and *A. niger* (4.2%) and the largest decrease in the networks of *M. barkeri* (35.3%), *M. acetivorans* (11%), *C. reinhardtii* (9.9%) and *T. maritima* (9.6%).

## Conclusion

Here we introduced the notion of effective deficiency that takes into account not only the structure of the network but also its operational constraints in the calculation of deficiency. The effective deficiency relies on the presence of nonstoichiometric balanced complexes, which we have shown to be present in large-scale metabolic networks across kingdoms of life. In addition, our results point at a subtle relation between effective deficiency and robustness of some reaction fluxes. Future work will aim at employing these findings in characterizing classes of networks that exhibit particular dynamical properties that can be ensured or precluded based on the notion of effective deficiency.

### Supplementary Information

Supplementary Information.

## Data Availability

The code, as well as all networks used in the analysis are available here.
